# Overeducation, vertical skill mismatch, and employment outcomes among Chinese university graduates: the mediating role of skill utilization

**DOI:** 10.3389/fpsyg.2026.1859874

**Published:** 2026-06-24

**Authors:** Yawen Yin, Cong Wang

**Affiliations:** 1 Foreign Language College, Weifang University, Weifang, China; 2 College of Elementary Education, Hainan Normal University, Haikou, China

**Keywords:** graduate employability, higher education, overeducation, skill utilization, vertical skill mismatch

## Abstract

This study examines how overeducation and vertical skill mismatch jointly shape graduates’ employment experiences in China’s rapidly expanding higher education system. It also explores whether skill utilization functions as a behavioural mechanism linking educational mismatch to employment satisfaction and employment quality. Grounded in human capital theory, job–skill matching theory, and social cognitive career theory, the study integrates structural and psychological perspectives into a unified framework. A quantitative survey was conducted with 512 recent graduates from 15 Chinese universities, and a serial mediation structural equation model (SEM) was used to examine the indirect associations of overeducation with employment outcomes through vertical skill mismatch and skill utilization. The results suggest that overeducation is significantly associated with a higher likelihood of vertical skill mismatch, which is in turn related to fewer opportunities for skill utilization. Lower skill utilization is further associated with lower employment satisfaction and employment quality, providing support for a sequential mediation pathway. These findings are consistent with the view that the consequences of higher education expansion may operate through interrelated structural and behavioural mechanisms rather than through direct associations alone. By identifying skill utilization as a key mechanism, the study contributes to graduate employability research and offers practical implications for curriculum design, career guidance, and labour market policy in China.

## 1 Introduction

In recent years, the rapid expansion of higher education has substantially increased the number of university graduates entering the labour market, intensifying competition for high-quality employment opportunities. In China, where the gross enrollment rate in higher education surpassed 50% in 2019—marking the universalization of tertiary education—the number of college graduates reached a record 11.58 million in 2023 (Xiang et al., 2023). While this expansion has contributed to the accumulation of human capital and enhanced opportunities for social mobility, it has also generated pronounced structural mismatches between educational attainment and labour market demand, manifested in phenomena such as overeducation, vertical skill mismatch, and underemployment (Zhou, 2023).

A growing body of research has documented the adverse consequences of overeducation and vertical skill mismatch for graduates’ employment outcomes, including wage penalties, reduced job satisfaction, and constrained career development (Ma et al., 2022). Overeducation—defined as a situation in which individuals’ educational attainment exceeds the formal requirements of their jobs—has become increasingly prevalent among Chinese graduates due to the rapid expansion of university enrollment combined with limited growth in high-skill employment opportunities (Zheng et al., 2021). Closely related to overeducation, vertical skill mismatch restricts graduates’ opportunities to deploy their acquired competencies effectively in the workplace, often resulting in the underutilization of human capital and diminished employment satisfaction (Jin and Peng, 2025).

Beyond its structural dimensions, educational and vertical skill mismatch has also been associated with a range of psychological experiences, such as employment anxiety and reduced career self-efficacy, particularly among young graduates navigating highly competitive labour markets (Zeng et al., 2021). These psychological responses highlight that mismatch is not merely an allocative inefficiency but also a lived employment experience. However, while prior research has emphasized emotional and cognitive reactions to mismatch, considerably less attention has been paid to the behavioural mechanisms through which structural mismatch is translated into concrete employment outcomes.

Several important gaps therefore remain in the existing literature. First, much of the research on overeducation and vertical skill mismatch has focused on economic outcomes such as wages and productivity, with relatively limited examination of the intermediate mechanisms linking mismatch to subjective employment experiences. Second, existing empirical evidence is heavily concentrated in Western labour markets, leaving the mechanisms operating within China’s rapidly expanding and structurally transforming graduate labour market insufficiently explored (Zheng, 2025). Third, although psychological constructs such as employment anxiety and career self-efficacy have been widely discussed, comparatively fewer studies have examined how mismatch constrains graduates’ ability to actively utilize their skills at work, thereby shaping employment satisfaction and employment quality.

To address these gaps, the present study focuses on skill utilization as a key behavioural mechanism linking educational and vertical skill mismatch to employment outcomes among Chinese university graduates. Drawing on human capital theory, job–skill matching theory, and social cognitive career theory, this study conceptualizes skill utilization as the extent to which graduates are able to apply and develop their acquired skills in their current jobs. Methodologically, the study adopts a quantitative survey design involving recent graduates from diverse disciplines and universities across China and employs structural equation modelling (SEM) to test a serial mediation framework linking overeducation, vertical skill mismatch, skill utilization, and employment outcomes. By integrating structural mismatch theory with a behavioural mechanism, this study advances research on graduate employability, extends existing mismatch research beyond psychological reactions to the functional deployment of skills, and provides empirical evidence from China that enriches understanding of graduate employment dynamics in non-Western labour markets characterized by rapid higher education expansion.

The contribution of this study should therefore be understood as mechanism-oriented rather than prevalence-oriented. While large-scale labour force surveys are valuable for estimating the incidence and wage consequences of educational mismatch, they often provide limited information about the behavioural processes through which mismatch is translated into graduates’ subjective and multidimensional employment outcomes. By focusing on skill utilization, this study examines how perceived overeducation and vertical skill mismatch are associated with graduates’ opportunities to apply their acquired competencies at work, and how this process is further related to employment satisfaction and employment quality. In this sense, the value of the present sample lies not in national representativeness, but in its ability to support a theoretically grounded analysis of the mismatch–utilization–outcome pathway among recent Chinese university graduates.

## 2 Literature review

### 2.1 Educational mismatch, overeducation, and skill mismatch: conceptual clarifications

Educational mismatch is an umbrella concept referring to a misalignment between individuals’ educational background and the requirements or content of their jobs. It is commonly distinguished into vertical and horizontal forms. Vertical educational mismatch concerns the level of education required by a job relative to the worker’s attained education, and includes both overeducation, where educational attainment exceeds job requirements, and undereducation, where educational attainment falls below job requirements (Verhaest and Omey, 2006; McGuinness et al., 2018). Horizontal mismatch, by contrast, refers to a mismatch between an individual’s field of study and the substantive content of the job (Robst, 2007; Somers et al., 2019).

In the present study, the focus is specifically on perceived overeducation as one form of vertical educational mismatch among university graduates. Perceived overeducation captures graduates’ own assessments of whether their educational attainment exceeds the requirements of their current jobs, and is particularly relevant for examining work attitudes and employment experiences (Verhaest and Omey, 2006; Arvan et al., 2019). Undereducation is conceptually acknowledged but not empirically examined, because the study is concerned with graduates whose educational credentials exceed rather than fall below job requirements. Skill mismatch is treated as a related but conceptually distinct construct. Whereas educational mismatch concerns the level or field of formal education, skill mismatch concerns the alignment between workers’ actual skills and the skills required in their jobs (Allen and Van der Velden, 2001; Flisi et al., 2017). Skill mismatch may also be vertical, involving surplus or deficient skills, or horizontal, involving a mismatch between skill type and job content. In the SEM analysis, skill mismatch refers primarily to vertical skill mismatch, operationalized as the discrepancy between graduates’ self-assessed skills and job-required skills. Horizontal mismatch is retained as a descriptive and subgroup variable rather than as a mediator in the structural model.

From a theoretical perspective, overeducation should not be conflated with skill mismatch. Rather, the two constructs are conceptually distinct but empirically related: overeducated graduates may be more likely to experience surplus skills when their jobs require lower levels of competence than those acquired through higher education. Some studies have interpreted overeducation within the frameworks of human capital theory and job–skill matching theory. Human capital theory posits that education enhances individual productivity and is expected to yield corresponding economic returns in the labour market. However, when the growth of educational supply significantly outpaces the demand for skills required by available jobs, a portion of job seekers are compelled to accept positions that require lower levels of education or skills than they possess. This situation directly leads to inefficiencies in the allocation of human capital (Allen and Van der Velden, 2001; Flisi et al., 2017).

Against this backdrop, job–skill matching theory further emphasizes that mismatches at the educational level are often accompanied by deeper mismatches in skills, whereby individuals’ skill levels or skill structures fail to align with actual job requirements (Allen and Van der Velden, 2001). The literature typically distinguishes between vertical skill mismatch and horizontal skill mismatch. Vertical skill mismatch refers to situations in which an individual’s skill level exceeds or falls short of job requirements, whereas horizontal skill mismatch highlights discrepancies between individuals’ fields of expertise or professional backgrounds and the content of their jobs. Although overeducation is not conceptually identical to skill surplus, a substantial body of empirical research suggests a stable and significant association between the two. In particular, under conditions of rapid expansion in higher education, highly educated graduates are more likely to enter jobs with relatively low skill requirements, thereby exhibiting pronounced vertical skill mismatch (Flisi et al., 2017; Rohrbach-Schmidt and Tiemann, 2016).

In sum, overeducation represents not only a form of structural mismatch at the educational level but also a mechanism through which mismatches between jobs and skills are intensified, with lasting effects on individuals’ work experiences and employment outcomes. This perspective provides an important basis for understanding the consequences of overeducation from a skill-mismatch perspective and sets the stage for subsequent analyses of how vertical skill mismatch shapes employment outcomes. Although a growing body of literature has examined overeducation and vertical skill mismatch in different national contexts, existing studies have not sufficiently explored these mechanisms in the Chinese context, nor how structural and behavioural factors jointly shape employment outcomes. Addressing this gap, the present study develops an integrated framework that connects structural mismatch theory with behavioural concepts of skill utilization.

### 2.2 Vertical skill mismatch, skill utilization, and employment outcomes

Under conditions of vertical skill mismatch, job seekers’ human capital is constrained in its effective deployment within their positions. Consequently, the association between vertical skill mismatch and employment outcomes may be partly explained by differences in skill utilization (Rohrbach-Schmidt and Tiemann, 2016). In recent years, a growing body of research has conceptualized skill utilization as a critical intermediary between job requirements and individual capabilities, emphasizing that the degree to which skills are applied plays a central role in shaping employment experiences and outcomes (García-Aracil and Van der Velden, 2008). When jobs fail to provide tasks and career development opportunities commensurate with individuals’ levels of ability, even highly educated or highly skilled workers may experience the devaluation or underutilization of their competencies, resulting in reduced skill utilization at work. Empirical studies indicate that vertical skill mismatch is frequently associated with lower levels of skill utilization, reflecting not only the waste of abilities under conditions of skill surplus but also functional misalignment arising from discrepancies between workers’ skill profiles and job content (Allen and Van der Velden, 2001; Flisi et al., 2017). Under such circumstances, workers often find it difficult to fully demonstrate their capabilities through their jobs, and their skill advantages are less likely to translate into actual performance or career-related returns (McGuinness et al., 2018).

Lower skill utilization, in turn, is associated with less favourable employment outcomes. Specifically, inadequate use of skills undermines workers’ perceptions of job competence and career accomplishment, leading to lower levels of overall job satisfaction (Verhaest and Omey, 2006; Arvan et al., 2019). Moreover, persistent skill underutilization may constrain career development trajectories, adversely affecting income levels, job stability, and long-term employment quality (Flisi et al., 2017; Rohrbach-Schmidt and Tiemann, 2016). By contrast, when individuals are able to fully apply and develop their skills in the workplace, they tend to report more positive work experiences and higher employment quality (McGuinness et al., 2018). Taken together, these findings suggest that vertical skill mismatch is associated with employment outcomes partly through lower skill utilization. This pathway-based perspective provides a more refined framework for understanding the employment consequences of vertical skill mismatch and overeducation, and offers a solid theoretical foundation for modelling the pathways linking vertical skill mismatch, skill utilization, and employment outcomes.

### 2.3 International evidence on overeducation and vertical skill mismatch

Recent studies suggest that overeducation and vertical skill mismatch are not unique to China but represent a broader challenge across both advanced and emerging labour markets. In European contexts, overeducation has been shown to vary across countries and regions, with recent evidence indicating that gender, care responsibilities, migration status, and regional labour-market conditions shape individuals’ risk of being overeducated (Baran, 2024). Cross-country evidence further shows that educational mismatch is associated with wage penalties, although the magnitude of these penalties differs substantially across institutional contexts, suggesting that labour-market flexibility, vocational pathways, and opportunities for mobility condition the consequences of mismatch (Sun, 2025). These studies indicate that mismatch should be understood not only as an individual-level employment problem but also as an institutional outcome shaped by the interaction between higher education systems and labour-market structures.

Evidence from developing and emerging economies further highlights the relevance of this issue beyond Western labour markets. In countries undergoing higher education expansion and economic restructuring, graduate mismatch often reflects a simultaneous oversupply of degree holders and insufficient growth in high-skill employment opportunities. For example, recent research on Morocco and Serbia shows that graduate labour markets may experience both overqualification and underskilling, suggesting that expanding higher education alone does not guarantee effective skill formation or labour-market absorption (Prica et al., 2025). Similarly, evidence from Vietnam shows that job–education mismatch among graduates has heterogeneous wage implications, with mismatch disadvantaging lower-wage graduates while benefiting some graduates in higher wage positions (Nguyen et al., 2026). These findings suggest that the consequences of mismatch in emerging economies are not uniform but depend on occupational structure, field of study, and labour-market segmentation.

Recent Chinese evidence further confirms the relevance of this issue in the context of rapid higher education expansion. Studies have documented substantial overeducation among Chinese graduates and its association with wage and job-satisfaction penalties (Zhang et al., 2024; Jones et al., 2025). More recent evidence also indicates that the wage effects of educational mismatch in China vary across career stages and gender groups, suggesting that work experience and labour-market structure shape the consequences of overeducation over time (Jiang et al., 2026). In addition, cohort-based evidence shows that China’s higher education expansion has produced persistent cohort-level effects on overeducation, income returns, and group disparities, further highlighting the structural nature of education–employment mismatch (Zhang et al., 2026).

Taken together, this literature suggests that overeducation and vertical skill mismatch have both general and context-specific dimensions. Across countries, mismatch is commonly associated with lower earnings, reduced job satisfaction, and inefficient use of human capital. However, the mechanisms through which mismatch affects employment outcomes may differ depending on the pace of higher education expansion, the availability of high-skilled jobs, and institutional pathways between education and work. In China, the rapid expansion of higher education, intense graduate competition, and uneven upgrading of occupational structures make it especially important to examine not only whether graduates are mismatched, but also how mismatch limits the actual use of their skills at work. This provides the rationale for the present study’s focus on skill utilization as a behavioural mechanism linking overeducation and vertical skill mismatch to employment satisfaction and employment quality.

### 2.4 Research hypotheses

Based on the preceding discussion of overeducation, vertical skill mismatch, and skill utilization, this study develops an analytical framework to examine the mechanisms through which overeducation is associated with employment outcomes. Specifically, overeducation is expected to be associated with higher levels of vertical skill mismatch, which in turn constrains skill utilization. Reduced skill utilization is further expected to be negatively associated with employment satisfaction and employment quality. Accordingly, the following hypotheses are proposed:

*H1:* Perceived overeducation is positively associated with vertical skill mismatch.

*H2:* Vertical skill mismatch is negatively associated with skill utilization.

*H3a:* Skill utilization is positively associated with employment satisfaction.

*H3b:* Skill utilization is positively associated with employment quality.

*H4:* Vertical skill mismatch and skill utilization jointly mediate the association between perceived overeducation and employment outcomes.

Building on human capital theory’s emphasis on education–productivity relationships and job–skill matching theory’s focus on alignment between skills and work tasks, this study integrates structural and behavioural perspectives to examine how educational and vertical skill mismatches jointly affect employment outcomes. By further incorporating insights from social cognitive career theory, it conceptualizes skill utilization as a behavioural mechanism that connects structural mismatches to graduates’ subjective experiences of employment quality.

While prior research has examined skill utilization as a predictor of job satisfaction or performance in Western labour markets, limited attention has been paid to its role as a behavioural mechanism mediating the effects of structural mismatches in the context of China’s mass higher education system. This study advances the conceptualization of skill utilization by embedding it within a serial mediation framework specifically tailored to the Chinese graduate labour market, where rapid expansion and structural misalignment have become increasingly pronounced.

Structural factors are grounded in Human Capital Theory and Job–Skill Matching Theory, while the behavioural mechanism of skill utilization is informed by Social Cognitive Career Theory.

Hypotheses H1–H4 represent the sequential mediation structure tested in the study.

**Figure 1:**
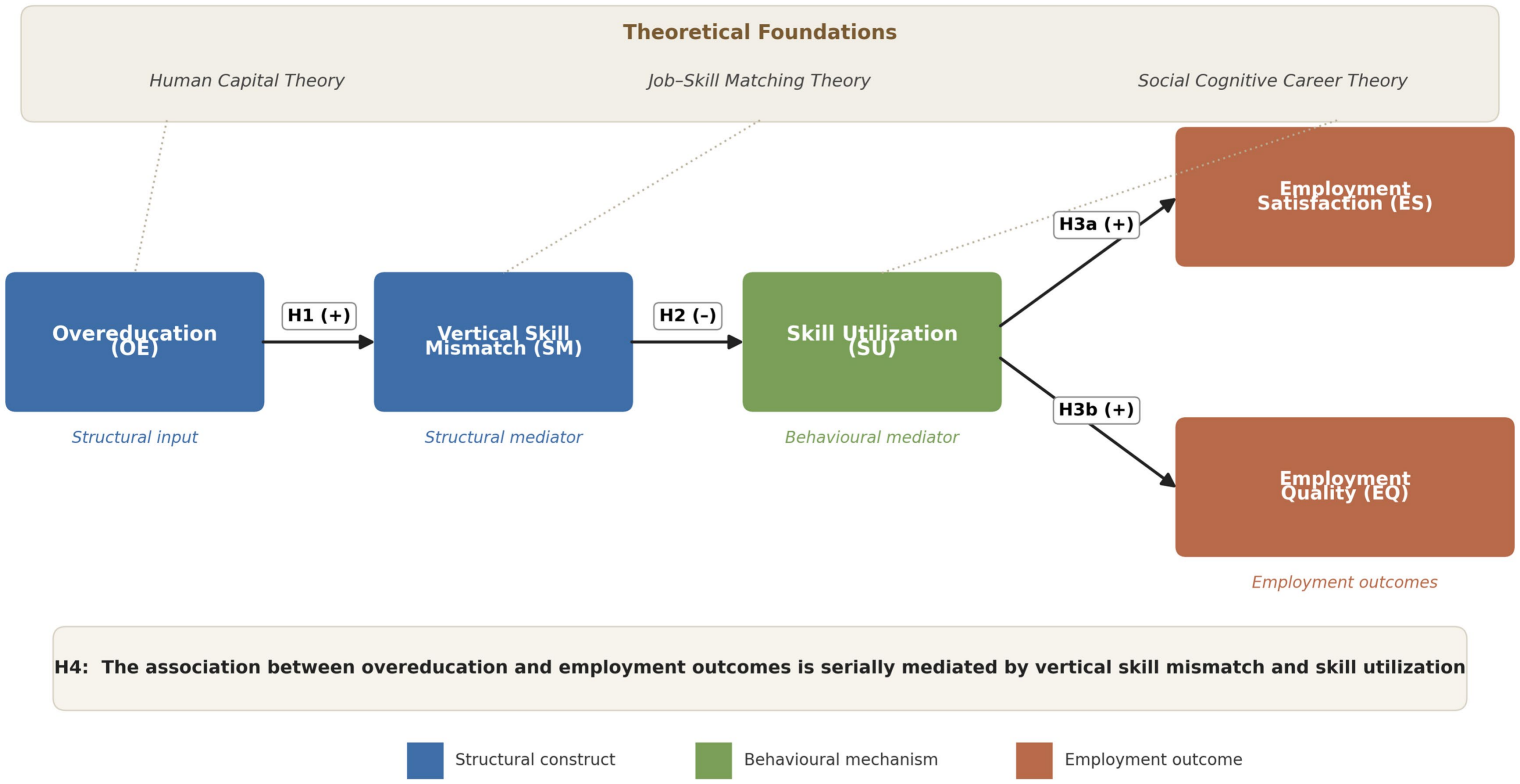
Theoretical framework and proposed serial mediation model. The model illustrates the hypothesized pathways linking overeducation to employment outcomes through vertical skill mismatch and skill utilization.

As illustrated in Figure 1, the proposed framework depicts a serial mediation model in which overeducation influences employment outcomes via vertical skill mismatch and skill utilization. By integrating structural determinants and behavioural mechanisms, this model captures the multi-level dynamics underlying graduate employability. It also provides the analytical foundation for the empirical investigation presented in the next section.

## 3 Methodology

### 3.1 Research design

This study adopted a quantitative, cross-sectional survey design to investigate the associations (rather than causal effects) between overeducation, vertical skill mismatch, and employment outcomes among Chinese university graduates. Guided by human capital theory and job-skill matching theory, the study employed validated scales to measure self-reported vertical skill mismatch, perceived overeducation, and employment satisfaction. This design allows for the examination of both the prevalence and the consequences of mismatch phenomena in the graduate labour market. Although causal inferences cannot be definitively established due to the cross-sectional design, the proposed relationships follow a theoretically plausible temporal order, insofar as educational attainment is typically completed prior to entry into the labour market and subsequent employment outcomes.

### 3.2 Participants

Participants were 512 recent graduates who had completed their university studies within the previous three years. They were recruited from 15 universities located in eastern, central, and western China. The sample included institutions with different disciplinary profiles and institutional types, including comprehensive universities, normal universities, finance and economics universities, and engineering-oriented or applied universities. This design was intended to increase contextual diversity across regions, institutional types, and fields of study, although the study does not claim national representativeness.

A two-stage non-probability sampling strategy was adopted, combining convenience sampling with snowball sampling. In the first stage, convenience sampling was used to recruit initial respondents through university alumni networks, graduate employment-related online groups, and personal academic contacts. These initial respondents were recent graduates known to meet the study’s eligibility criteria, namely having completed university studies within the previous three years and having experience of entering or seeking entry into the labour market. In the second stage, snowball sampling was implemented by asking eligible respondents to forward the online survey link to other recent graduates in their social, academic, or employment-related networks who also met the inclusion criteria. The survey invitation clearly stated the eligibility requirements, and responses that did not meet these criteria or had invalid answers to the core variables were excluded.

Several steps were taken to reduce potential sampling bias. First, recruitment was conducted through multiple channels rather than a single university or contact group. Second, the survey was distributed across alumni networks and graduate employment-related groups from different regions and institutional types. Third, the final sample was checked to ensure that no single university dominated the dataset; the number of respondents from each institution ranged from 29 to 42. Fourth, the manuscript explicitly acknowledges that the sample is based on non-probability sampling and should not be interpreted as nationally representative. Instead, the sample is used to examine theoretically grounded associations among perceived overeducation, vertical skill mismatch, skill utilization, and employment outcomes among recent Chinese graduates.

Participation was voluntary and anonymous, and informed consent was obtained before the questionnaire began.

The final sample covered graduates from 15 universities across three broad regions of China. Eastern China accounted for 205 respondents, Central China for 161 respondents, and Western China for 146 respondents. Detailed information on the participating universities, regional distribution, institutional type, number of respondents, and percentage of the total sample is provided in Supplementary material.

The sample covered diverse academic backgrounds, including engineering (29%), economics and management (23%), humanities and social sciences (31%), and natural sciences (17%). Approximately 54.3% of the respondents identified as female, and the mean age was 23.8 years (SD = 1.2). Although the use of non-probability sampling limits the generalizability of the findings, the inclusion of multiple universities across different regions and institutional types provides a useful basis for examining the associations among overeducation, vertical skill mismatch, skill utilization, and employment outcomes among recent Chinese graduates.

Although the sample is not nationally representative, it is informative for the purpose of this study for three reasons. First, all respondents were recent university graduates who had completed their studies within the previous three years, making them directly relevant to the school-to-work transition stage in which overeducation and skill mismatch are particularly salient. Second, the sample covers graduates from eastern, central, and western China as well as different institutional types and disciplinary backgrounds, allowing the study to capture variation across educational and regional contexts. Third, the survey collected construct-level information on perceived overeducation, vertical skill mismatch, skill utilization, employment satisfaction, and employment quality, which are rarely available together in large administrative or labour force datasets. Therefore, while the sample should not be used to estimate national prevalence rates, it provides an appropriate empirical basis for examining the proposed mechanism among recent Chinese graduates.

### 3.3 Instruments

The instruments were organized according to the core theoretical constructs specified in the proposed serial mediation model rather than the number of analytical dimensions. Specifically, perceived overeducation, vertical skill mismatch, skill utilization, and employment outcomes were treated as distinct constructs corresponding to different stages of the education–employment process. Vertical and horizontal mismatch were operationalized as complementary dimensions of the broader construct of skill mismatch, while employment satisfaction and employment quality were conceptualized as indicators within the employment outcome domain.

The questionnaire used in this study was developed by adapting items and measurement frameworks from previously published instruments and by constructing additional indicators for the employment quality composite. To improve measurement transparency, Supplementary material reports the adapted questionnaire information used in the present study, including item codes, construct labels, response scales, coding procedures, aggregation rules, and measurement sources. Because several measures were adapted from previously published instruments, the appendix reports the adapted items and coding information used in this study rather than reproducing the original published instruments verbatim.

Unless otherwise stated, all scale items were measured on a 5-point Likert scale (1 = strongly disagree, 5 = strongly agree).

#### 3.3.1 Perceived overeducation

Perceived overeducation was measured using three adapted items based on Verhaest and Omey (2006). The adapted items assessed the extent to which respondents perceived their current job as requiring qualifications lower than their educational level. The scale demonstrated acceptable internal consistency in the present study (Cronbach’s *α* = 0.84).

#### 3.3.2 Skill mismatch

Skill mismatch was assessed primarily in terms of vertical skill mismatch using adapted multi-item measures based on Allen and Van der Velden (2001). Participants reported both the level of skills required by their current job and their self-assessed skill levels across four domains: analytical ability, communication, teamwork, and problem solving. For each domain, a discrepancy score was calculated by comparing self-assessed skill level with job-required skill level. These domain-specific discrepancy indicators were used to represent the latent construct of vertical skill mismatch in the SEM. Higher scores indicated greater surplus skills. The measure demonstrated high reliability in this study (Cronbach’s α = 0.87).

In addition, horizontal mismatch was captured by a categorical item indicating whether the participant’s current job was related to their field of study (matched vs. mismatched). Together, vertical and horizontal mismatch were treated as complementary dimensions of the broader construct of skill mismatch.

Although skill mismatch can include both vertical and horizontal dimensions, the present SEM analysis focuses on vertical skill mismatch, which captures the degree of surplus skills and is more suitable for continuous path modelling. Horizontal mismatch was retained for descriptive subgroup analyses comparing horizontally matched and mismatched graduates on key study variables.

Because the study aims to examine the employment consequences of surplus education and surplus skills among recent university graduates, undereducation and skill deficit are not modelled in the SEM. This scope limitation is acknowledged when interpreting the findings.

#### 3.3.3 Skill utilization

Skill utilization was measured using adapted items based on García-Aracil and Van der Velden (2008). The adapted items assessed the extent to which graduates were able to use and further develop their acquired skills in their current jobs. The scale demonstrated good internal consistency in the present study (Cronbach’s *α* = 0.87).

#### 3.3.4 Employment outcomes

Employment outcomes were operationalized using two complementary constructs: employment satisfaction and employment quality.

Employment satisfaction was measured using five adapted items based on Brayfield and Rothe’s (1951) job satisfaction index. The adapted items assessed graduates’ overall affective and evaluative judgments about their current job and demonstrated excellent reliability in this study (Cronbach’s α = 0.90). Because the scale was adapted from a previously published instrument, Supplementary material reports the item codes, construct information, response scale, and coding procedure used in the present study rather than reproducing the original published scale items verbatim.

Whereas employment satisfaction reflects graduates’ subjective evaluations of their work experiences, employment quality was used to capture more objective and externally observable aspects of graduates’ employment conditions. Following the logic of large-scale graduate employment surveys in China, employment quality was operationalized as a composite observed outcome rather than as a latent psychological construct. Four indicators were selected because they represent commonly used and substantively distinct dimensions of graduate employment quality: monthly income, job stability, employment status, and major–job match. Monthly income captures the economic return of employment; job stability reflects perceived security and continuity of the current position; employment status indicates the degree of labour market attachment; and major–job match reflects the relevance between graduates’ field of study and current work.

Each indicator was coded so that higher values represented better employment quality. Monthly income was coded on a five-point ordinal scale, ranging from lower to higher income bands. Job stability was measured on a five-point scale from very unstable to very stable. Employment status was recoded as an ordered indicator, with higher values representing more secure or regular employment status. Major–job match was coded on a five-point scale, ranging from not related at all to highly related. To make the four indicators comparable, all indicators were transformed onto the same 1–5 metric before aggregation. The composite employment quality index was then calculated as the average of the four indicators, with higher scores indicating better employment quality. Full coding information and aggregation procedures for the employment quality composite are provided in Supplementary material.

Equal weighting was used for two reasons. First, the four indicators represent complementary dimensions of employment quality rather than interchangeable reflections of a single latent factor. There was therefore no strong theoretical basis for assigning one dimension greater weight than the others. Second, equal weighting avoids imposing arbitrary assumptions about the relative importance of income, stability, employment status, and major–job match. The index was therefore treated as a formative composite observed variable rather than as a reflective latent construct. In other words, the indicators were assumed to jointly constitute employment quality, rather than to reflect an underlying psychometric scale.

The potential conceptual overlap between major–job match and horizontal mismatch was also considered. Horizontal mismatch refers to the descriptive condition in which a graduate’s current job is or is not related to their field of study. In the present study, however, horizontal mismatch was not included in the structural equation model as a mediator. The SEM analysis focused on vertical skill mismatch, which captures the degree of surplus skills and is more suitable for continuous path modelling. Major–job match was retained as one component of the employment quality composite because it reflects an employment outcome condition rather than a mechanism in the structural pathway. This distinction reduces the risk of conceptual circularity between the mediator and the outcome variable.

### 3.4 Procedure

The online survey was administered through the Wenjuanxing platform. Prior to data collection, two experts in education and labour economics evaluated the questionnaire to ensure content validity and cultural appropriateness. A pilot study involving 36 graduates was conducted to assess item clarity and preliminary reliability, resulting in minor refinements to wording. Participation was voluntary and anonymous, and informed consent was obtained electronically. The final survey required approximately 20 min to complete. Data collection took place from March to April 2025. Unemployed and job-seeking respondents were instructed to answer job-related items with reference to their most recent employment experience. This instruction was intended to provide a consistent reference point for job-related measures such as overeducation, vertical skill mismatch, skill utilization, employment satisfaction, and job stability. At the same time, respondents’ current employment status was retained as one component of the employment quality composite, allowing the analysis to reflect differences in current labour market attachment without excluding unemployed or job-seeking graduates from the sample.

For measures adapted from English-language sources, a translation and contextual adaptation procedure was conducted before the formal survey. The research team first reviewed the original measurement sources and adapted the items for the Chinese graduate employment context. The adapted items were translated into Chinese by bilingual researchers familiar with higher education and graduate employment research. The Chinese version was then reviewed for semantic clarity, conceptual equivalence, and contextual appropriateness. After data collection, the reliability and validity of the multi-item measures were evaluated using Cronbach’s alpha, confirmatory factor analysis, composite reliability, AVE, and discriminant validity tests.

### 3.5 Data analysis

Data analyses were conducted using SPSS 29 and Mplus 8.0. Prior to hypothesis testing, all variables were screened for missing data, outliers, and normality. Common method bias was tested using Harman’s single-factor test, and no single factor accounted for more than 40% of the variance, suggesting that common method variance was not a serious concern. Additionally, a confirmatory single-factor model showed poor fit (CFI < 0.70, RMSEA > 0.15), providing further evidence that common method bias was minimal. Descriptive statistics (mean, standard deviation, and frequency) were computed for all study variables, including overeducation, skill mismatch (vertical and horizontal), skill utilization, employment satisfaction, and employment quality. For the employment quality composite, the coding direction of all four indicators was checked before aggregation so that higher scores consistently indicated better employment quality. The composite was treated as an observed formative index rather than a reflective latent scale; therefore, internal consistency statistics such as Cronbach’s alpha were not used as the primary criterion for evaluating its validity. Moreover, the hypothesized serial mediation model involves multiple theoretically ordered constructs, making it less likely that the observed relationships can be attributed to a single common method factor.

Pearson correlation analyses were performed to examine the bivariate relationships among continuous variables, while point-biserial correlations were used for dichotomous variables. To provide a more meaningful descriptive analysis of horizontal mismatch, additional subgroup comparisons were conducted between horizontally matched and horizontally mismatched graduates. Welch’s two-sample t tests were used because equal variances were not assumed, and Cohen’s d was reported to indicate the magnitude of group differences. Multicollinearity was assessed using variance inflation factors (VIF < 5). Subsequently, confirmatory factor analyses (CFA) were conducted to assess the construct validity of all multi-item measures, with acceptable fit indices (CFI > 0.95, RMSEA < 0.06, SRMR < 0.06).

Hierarchical multiple regression analyses were conducted in four steps to test the predictive effects of overeducation and vertical skill mismatch on employment outcomes. Control variables (gender, age, major, university type) were entered in Step 1, overeducation in Step 2, vertical skill mismatch in Step 3, and skill utilization in Step 4. Changes in R^2^ and standardized regression coefficients (*β*) were reported at each step. These analyses served to establish baseline associations among the study variables and to assess the incremental explanatory power of overeducation, vertical skill mismatch, and skill utilization.

SEM was subsequently employed to test the integrated theoretical framework and the hypothesized serial mediation mechanism. Specifically, SEM was used to estimate the integrated path model linking overeducation → vertical skill mismatch → skill utilization → employment outcomes. Given the heterogeneous nature of the constructs, the SEM was specified as a hybrid model combining latent variables and an observed composite outcome. Perceived overeducation, vertical skill mismatch, skill utilization, and employment satisfaction were modelled as latent variables measured by their respective multi-item indicators, whereas employment quality was modelled as an observed composite outcome. Employment quality was treated as a composite observed variable because its indicators represent distinct and complementary employment conditions rather than interchangeable reflections of a single underlying psychological construct. Model fit was evaluated using the comparative fit index (CFI) and Tucker–Lewis index (TLI), with values above 0.90 indicating acceptable fit, and the root mean square error of approximation (RMSEA) and standardized root mean square residual (SRMR), with values below 0.08 indicating acceptable fit. Bootstrapping with 5,000 resamples was used to test the significance of indirect effects.

An alternative reversed-order structural model was also tested to examine potential endogeneity or reverse ordering between vertical skill mismatch and skill utilization. The proposed model demonstrated good fit and was retained on theoretical grounds. Robustness checks further included multi-group analyses across gender, major categories, and university tiers to ensure the stability of the results.

## 4 Results

### 4.1 Descriptive statistics

A total of 512 participants completed the survey. Of these, 54.3% were female, and the mean age was 23.8 years (SD = 1.2). Regarding employment status, 62.5% were employed, 23.6% were seeking employment, and 13.9% were unemployed.

Descriptive statistics for the main study variables are presented in Table 1. The average overeducation score was M = 3.20, SD = 0.87, with 48.2% of participants reporting that their current job required lower qualifications than their educational attainment. Vertical skill mismatch averaged M = 2.96, SD = 0.79, and 44.7% of participants reported working in positions unrelated to their field of study (horizontal mismatch). Skill utilization was moderate (M = 3.13, SD = 0.84). Employment satisfaction was slightly below the scale midpoint (M = 2.89, SD = 0.83), and employment quality averaged M = 2.91, SD = 0.89. These results suggest that recent graduates perceive a moderate degree of job mismatch and relatively low levels of satisfaction with their employment outcomes.

**Table 1:** Descriptive statistics of main variables.

Variable	Mean (M)	SD	Notes
Overeducation	3.20	0.87	48.2% report overeducation
Vertical skill mismatch	2.96	0.79	—
Horizontal mismatch	—	—	44.7% mismatch
Skill utilization	3.13	0.84	—
Employment satisfaction	2.89	0.83	—
Employment quality	2.91	0.89	Composite index

To further examine horizontal mismatch, subgroup comparisons were conducted between graduates whose jobs were related to their field of study and those whose jobs were not. The results are reported in Supplementary material, Table B11. Horizontally mismatched graduates reported significantly higher vertical skill mismatch than horizontally matched graduates (M = 3.08 vs. 2.87, *p* = 0.003). They also reported lower skill utilization (M = 2.96 vs. 3.26, *p* < 0.001), lower employment satisfaction (M = 2.75 vs. 3.01, *p* < 0.001), and lower employment quality (M = 2.56 vs. 3.19, *p* < 0.001). To address the potential overlap between horizontal mismatch and the major–job match component of the employment quality composite, an additional comparison was conducted using an employment quality score excluding the match component. The group difference remained significant (M = 2.66 vs. 3.02, *p* < 0.001), suggesting that the employment quality difference was not solely driven by the mechanical inclusion of major–job match in the composite index.

### 4.2 Correlation analysis

Pearson correlation coefficients are shown in Table 2. Overeducation was positively correlated with vertical skill mismatch (r = 0.30, *p* < 0.001) and negatively correlated with skill utilization (r = −0.27, *p* < 0.001). Vertical skill mismatch was negatively associated with employment satisfaction (r = −0.42, *p* < 0.001) and employment quality (r = −0.33, *p* < 0.001). Skill utilization showed a strong positive correlation with both employment satisfaction (r = 0.50, *p* < 0.001) and employment quality (r = 0.49, *p* < 0.001).

**Table 2:** Pearson correlation matrix of key study variables.

Variable	1	2	3	4	5
1 Overeducation	1	0.30***	−0.27***	−0.21***	−0.19***
2 Vertical skill mismatch	0.30***	1	−0.33***	−0.42***	−0.33***
3 Skill utilization	−0.27***	−0.33***	1	0.50***	0.49***
4 Employment satisfaction	−0.21***	−0.42***	0.50***	1	0.46***
5 Employment quality	−0.19***	−0.33***	0.49***	0.46***	1

### 4.3 Hierarchical regression analysis

Hierarchical regression results predicting employment satisfaction are summarized in Table 3.

Step 1: Demographic controls explained R^2^ = 0.08 of the variance.
Step 2: Adding overeducation increased ΔR^2^ = 0.04, with overeducation negatively predicting employment satisfaction (*β* = −0.20, *p* < 0.001).
Step 3: Adding vertical skill mismatch further increased variance explained (ΔR2 = 0.12), with vertical skill mismatch showing a stronger negative effect (β = −0.37, *p* < 0.001) than overeducation.
Step 4: Including skill utilization significantly improved model fit (ΔR^2^ = 0.12) and positively predicted employment satisfaction (*β* = 0.38, *p* < 0.001), indicating that skill utilization contributed additional explanatory power beyond demographic controls, overeducation, and vertical skill mismatch.

**Table 3:** Hierarchical regression results predicting employment satisfaction.

Step	Predictor	*β*	R^2^	ΔR^2^	*p*
Step 1	Controls (gender, age, major, univ. type)	—	0.08	—	< 0.001
Step 2	Overeducation	−0.20	0.12	0.04	< 0.001
Step 3	Vertical skill mismatch	−0.37	0.24	0.12	< 0.001
Step 4	Skill utilization	0.38	0.36	0.12	< 0.001

Full regression coefficients for all predictors and control variables, including standard errors, 95% confidence intervals, and model fit and comparison statistics (R^2^, adjusted R^2^, F, ΔR^2^, F-change, AIC, BIC) for both employment satisfaction and employment quality, are reported in Tables B1–B4 in the Supplementary material.

### 4.4 Structural equation modeling (SEM)

A SEM was estimated to examine the hypothesized sequential associations between overeducation, vertical skill mismatch, skill utilization, and employment outcomes. The proposed model specified a theoretically informed pathway in which overeducation was associated with higher levels of vertical skill mismatch, vertical skill mismatch was associated with lower skill utilization, and skill utilization was associated with employment outcomes. In this model, perceived overeducation, vertical skill mismatch, skill utilization, and employment satisfaction were estimated as latent variables, while employment quality was included as an observed composite outcome.

**Figure 2:**
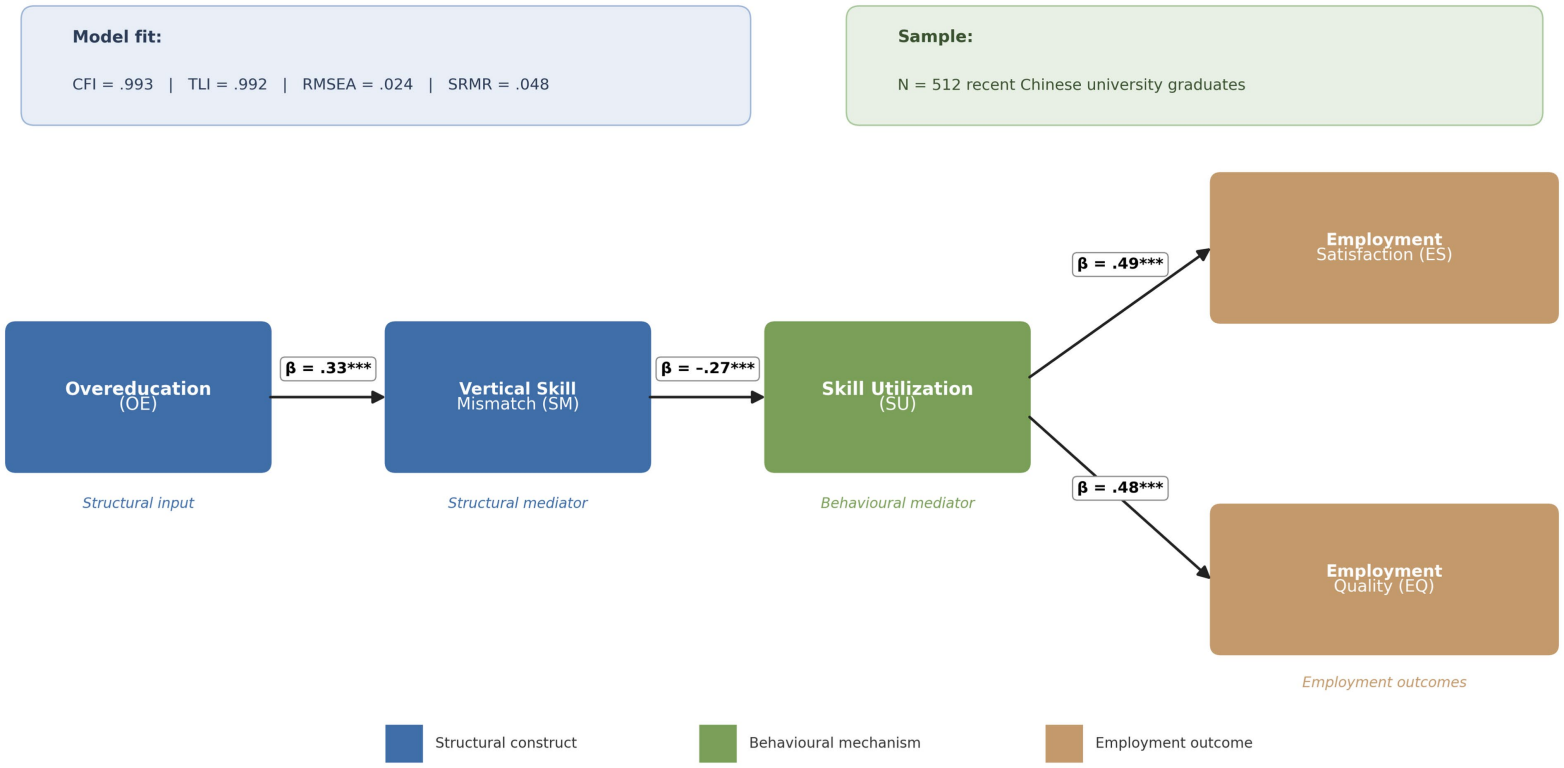
Structural equation model with standardized path coefficients. Solid arrows indicate the primary structural paths; the direct path from overeducation to skill utilization and direct residual paths from overeducation and vertical skill mismatch to employment outcomes were estimated but are omitted from the figure for clarity (see Table B8 for the full coefficient matrix). Model fit indices: CFI = 0.993, TLI = 0.992, RMSEA = 0.024, SRMR = 0.048. ***p < 0.001.

The overall model demonstrated good fit to the data: CFI = 0.993, TLI = 0.992, RMSEA = 0.024, and SRMR = 0.048, all of which meet commonly accepted thresholds for adequate model fit (Hu and Bentler, 1999). Consistent with theoretical expectations, overeducation was positively associated with vertical skill mismatch (β = 0.33, *p* < 0.001), and vertical skill mismatch was negatively associated with skill utilization (β = −0.27, *p* < 0.001). Skill utilization, in turn, was positively associated with employment satisfaction (β = 0.49, *p* < 0.001) and employment quality (β = 0.48, *p* < 0.001). These results provide support for the proposed mediation framework, suggesting that vertical skill mismatch and skill utilization are statistically consistent with a sequential pathway linking overeducation to employment outcomes. Bootstrapped indirect effect analysis further supported the proposed mediating role of vertical skill mismatch and skill utilization. Specifically, the serial indirect effect of overeducation on employment satisfaction through vertical skill mismatch and skill utilization (OE → SM → SU → ES) was statistically significant (B = −0.042, 95% CI [−0.064, −0.025], *p* < 0.001), and a parallel serial effect was observed for employment quality (B = −0.046, 95% CI [−0.069, −0.027], *p* < 0.001), providing statistical support for H4 (see Table B9 in the Supplementary material for the full decomposition). These results provide statistical evidence consistent with the serial mediation mechanism proposed in the theoretical model. Accordingly, Hypotheses H1 through H4 were supported, providing empirical support rather than causal proof for the theorized sequential mediation model. Standardized path coefficients are reported in Table 4. The full measurement model (factor loadings, CR, AVE, and discriminant validity), the estimation equations, the complete set of structural path coefficients with bias-corrected percentile bootstrap 95% confidence intervals (5,000 resamples), and the full decomposition of direct, indirect, and total effects — including the serial indirect pathway hypothesized in H4 — are reported in Tables B5–B9 in the Supplementary material. Nested-model comparisons against alternative specifications (parallel mediation, full mediation, and reversed mediator order) are reported in Table B10 (see Figure 2).

**Table 4:** Standardized path coefficients from the structural equation model.

Path	β	SE	*p*
Overeducation → Vertical skill mismatch	0.33	0.05	< 0.001
Overeducation → Skill utilization	−0.24	0.05	< 0.001
Vertical skill mismatch → Skill utilization	−0.27	0.05	< 0.001
Skill utilization → Employment satisfaction	0.49	0.05	< 0.001
Skill utilization → Employment quality	0.48	0.05	< 0.001
Overeducation → Employment satisfaction	0.00	0.05	0.968
Overeducation → Employment quality	0.01	0.05	0.902
Vertical skill mismatch → Employment satisfaction	−0.27	0.04	< 0.001
Vertical skill mismatch → Employment quality	−0.17	0.05	< 0.001

### 4.5 Robustness checks

A series of robustness checks were conducted to assess the stability and generalizability of the proposed structural model. Multi-group SEM analyses were performed across gender (male vs. female), disciplinary categories (engineering, economics/management, humanities/social sciences, natural sciences), and university type (high-tier vs. mid/low-tier institutions). Measurement of the latent constructs was first examined, and configural, metric, and scalar invariance models all demonstrated acceptable fit, indicating that the measurement structure was comparable across groups.

Subsequently, chi-square difference tests were conducted to compare constrained and unconstrained structural models. Across all grouping variables, the chi-square differences were nonsignificant (all *p* > 0.05), suggesting that the key structural paths in the proposed model did not differ systematically across gender, disciplinary background, or university type. These results support the overall robustness and stability of the structural relationships identified in the main analyses.

In addition, exploratory subgroup analyses indicated some variation in the magnitude of the sequential mediation effects across disciplinary fields. Specifically, the indirect pathway from overeducation to employment outcomes via vertical skill mismatch and skill utilization appeared to be relatively stronger among graduates in the humanities and social sciences. Although these differences did not reach statistical significance in formal multi-group comparisons, they suggest that field-specific skill structures may condition the strength of the mismatch–utilization relationship and warrant further investigation in future research.

## 5 Discussion

We investigated how overeducation, vertical skill mismatch, and skill utilization jointly shape the employment experiences of Chinese university graduates. Our results are consistent with a sequential pattern in which overeducation is associated with higher levels of vertical skill mismatch, which is in turn related to fewer opportunities for skill utilization and lower levels of employment satisfaction and employment quality. We emphasize that mismatches between education and job requirements are not isolated individual phenomena but reflect structural and institutional tensions between higher education expansion and labour market absorption capacity. In recent years, China’s higher education system has expanded at an unprecedented scale, with official estimates indicating around 12.2 million university graduates entering the labour market in 2025 and similar historic highs in the preceding years (Xinhua News Agency, 2024); at the same time, youth unemployment has remained elevated, with the urban jobless rate for those aged 16–24 reported at around 16.9 per cent in early 2025 (Wu, 2025). These dynamics highlight persistent labour market pressures and underscore the urgency of understanding how educational expansion interacts with labour market structures to shape graduate employment outcomes.

### 5.1 Discussion of hypotheses

Our findings supported Hypothesis 1, showing that overeducation is positively associated with vertical skill mismatch. This result suggests that the rapid expansion of higher education in China has generated an oversupply of degree holders whose educational qualifications exceed occupational requirements, leading to both vertical and horizontal mismatches between acquired and job-required skills. Such patterns reflect a structural imbalance between the production of human capital and the pace of labour market upgrading. Recent large-scale data analyses similarly indicate that overeducation has become a persistent rather than transitory phenomenon in the Chinese graduate labour market (Zheng et al., 2021). Together, these findings underscore that educational expansion, when not accompanied by structural economic transformation, tends to deepen rather than alleviate mismatch pressures.

We found strong support for Hypothesis 2, which proposed that vertical skill mismatch is associated with fewer opportunities for skill utilization. The data suggest that mismatched workers may be more likely to occupy positions that provide fewer opportunities to apply and develop their competencies, resulting in partial underuse of human capital. This pattern is consistent with research showing that when employees’ skills do not align with job requirements, their capacity for learning and productivity diminishes over time (Jin and Peng, 2025). The finding therefore highlights a crucial intermediary process: mismatch operates not only as an allocative inefficiency but also as a functional constraint that prevents education from being effectively transformed into productive outcomes.

The results further provide clear evidence for Hypotheses 3a, 3b, indicating that higher levels of skill utilization are associated with greater employment satisfaction and employment quality. Graduates who are able to fully employ and further develop their competencies report higher satisfaction and perceive their employment as more meaningful and sustainable. This finding resonates with earlier work emphasizing that opportunities for skill use are central to perceptions of job quality and subjective well-being (Ma et al., 2022). From this perspective, employment outcomes depend not only on whether individuals find jobs, but also on the extent to which those jobs allow the exercise of their accumulated human capital.

Finally, the analysis provided support for the sequential mediation pattern proposed in Hypothesis 4, indicating that the association between overeducation and employment outcomes is statistically mediated by vertical skill mismatch and subsequent skill utilization. The mediation results suggest that graduates’ overqualification is associated with a higher likelihood of mismatch, which in turn is related to fewer opportunities for skill use, ultimately relating to lower job satisfaction and employment quality. These findings lend empirical support to the proposed serial mediation framework and offer a pathway-based interpretation of overeducation. Notably, the direct effects of overeducation on employment satisfaction and employment quality were no longer statistically significant after vertical skill mismatch and skill utilization were included in the model. This pattern suggests that the proposed sequential pathway provides a plausible explanation for the observed associations. Together, these results deepen understanding of how education–employment imbalances are translated into differentiated employment experiences within China’s evolving knowledge economy.

### 5.2 Integration with existing literature and implications

Compared with large-scale studies that primarily estimate the prevalence, wage penalties, or distributional consequences of job–education mismatch, the present study contributes by opening the black box between mismatch and employment outcomes. In particular, it identifies skill utilization as a behavioural mechanism through which overeducation and vertical skill mismatch are associated with graduates’ employment satisfaction and employment quality. This mechanism is especially relevant in the Chinese context, where rapid higher education expansion has produced a large supply of degree holders, but the upgrading of occupational structures and high-skill job opportunities has been uneven. Under such conditions, the key issue is not only whether graduates are formally mismatched, but also whether their acquired skills can be meaningfully used in the workplace. By modelling this pathway, the study adds explanatory depth to existing mismatch research and complements, rather than competes with, large-scale labour force survey studies.

Although the cross-sectional design does not allow firm causal claims, the sequence specified in the model is theoretically grounded and is further supported by the good fit of the proposed model and by the theoretically grounded ordering of the constructs. Taken together, the findings support the integrated framework developed in this study, showing that graduates’ employment experiences are shaped not only by structural forms of mismatch but also by whether their skills can be meaningfully utilized at work.

This study contributes to the growing literature on graduate employability and educational mismatch by showing evidence consistent with continuing tensions between educational attainment and labour market demand in the context of higher education massification. Consistent with earlier research, overeducation and vertical skill mismatch remain persistent features of graduate labour markets, reflecting a misalignment between educational expansion and industrial upgrading (Sloane, 2014). While previous studies have often emphasized wage penalties or short-term employment effects, the present study extends this discussion by identifying skill utilization as a key mechanism through which mismatch affects employment satisfaction and employment quality. In contrast to findings from Western contexts, where mismatch may be alleviated through labour mobility, the persistence observed among Chinese graduates points to stronger institutional and structural constraints (Li et al., 2024). This is also in line with OECD-level evidence showing that educational and job mismatches often persist across advanced economies and are shaped by labour market institutions and credential norms that influence mobility and career progression (Sun, 2025).

Beyond situating the study within the existing literature, the findings provide several theoretical implications. They highlight the need to reinterpret human capital theory in functional rather than purely quantitative terms, education generates value only when graduates have the opportunity to apply and develop their skills in appropriate occupational contexts. The results also extend job–skill matching theory by showing how vertical skill mismatch is linked to employment outcomes through reduced skill utilization. Moreover, the conceptual relevance of psychological processes such as career self-efficacy and employment anxiety helps connect macro-level labour structures with individual-level behaviour, providing conceptual bridges between structural mismatch theory and social cognitive career frameworks. This synthesis deepens understanding of how institutional and psychological factors jointly determine employability in mass higher education systems.

The study also has practical implications. Universities should strengthen the alignment between academic curricula and occupational skill requirements through curricular reform, experiential learning, and closer industry-university collaboration (Müller, 2024). Career services should also take a more active role in addressing employment anxiety and strengthening career self-efficacy through tailored guidance, skill audits, and career simulations. At the same time, government agencies, higher education institutions, and employers need to work more closely to improve the match between graduate supply and changing labour market demand. In this sense, addressing overeducation and vertical skill mismatch is not simply a matter of expanding education, but of creating institutional conditions in which graduates’ skills can be properly recognized, developed, and used.

### 5.3 Cross-national contextual differences: comparing Europe and China

Although this study focuses on the Chinese labour market, comparing trends of overeducation and mismatch internationally helps contextualize the findings. In advanced economies, educational mismatch (where workers’ qualifications exceed job requirements) is a well-documented phenomenon associated with labour market structural imbalances and institutional factors such as the supply of tertiary graduates relative to labour demand and the relative strength of vocational pathways (Green and Henseke, 2016). For example, in comparative analyses across European Union countries, educational mismatch (including overeducation and undereducation) has been measured and tracked over time, showing variation in incidence across national contexts, and indicating that mismatch remains a persistent feature of EU labour markets even as educational attainment rises (McGuinness et al., 2017; Baran, 2024).

For China, prior studies document the existence of overeducation among graduates, but the reported prevalence is sensitive to measurement choices. For example, Li (2021) reported an overeducation estimate of approximately 35.09% for China’s urban labour market in 2015, based on an improved measurement approach comparing individuals’ educational attainment with occupational requirements. Zhang et al. (2024) later cited this estimate and further emphasized that overeducation can be measured through different approaches, including subjective self-assessment, objective job-analysis methods, statistical realised-matches methods, and occupation-based classifications. Therefore, the 35.09% figure should be interpreted as a measurement-specific estimate rather than as a definitive national prevalence rate. Recent graduate-focused evidence also suggests that estimated rates of overeducation in China vary across established measurement approaches, indicating that overeducation is a meaningful but measurement-sensitive issue in China’s graduate labour market (Jones et al., 2025; Zhang et al., 2024). This pattern reflects the rapid expansion of higher education in China over the past two decades without equivalent growth in high-skill employment, resulting in educational supply outpacing demand in several segments of the labour market.

Comparative studies of wage effects across OECD countries show that educational mismatch typically carries a wage penalty, with overeducated workers earning on average about 14% less than their matched counterparts, though penalties vary substantially across labour markets and institutional contexts (Sun, 2025). While similar cross-national wage penalty estimates are yet to be uniformly reported for China in official OECD publications, research on Chinese microdata indicates that overeducation is negatively associated with job satisfaction and wages, suggesting a penalty effect is present in Chinese labour markets as well (Ma et al., 2022).

Overall, both Europe and China face substantial educational mismatch among tertiary graduates, but the underlying dynamics and labour market consequences are not the same. In Europe, overeducation is often discussed in the context of high rates of tertiary attainment, changing supply–demand relations, and institutional differences across countries. Its prevalence varies, but it remains a persistent concern. In China, by contrast, rapid higher education expansion has produced a large cohort of degree holders entering a labour market that has not fully generated enough high-skill positions. This has contributed to meaningful levels of overeducation among recent graduates, together with noticeable penalties in wages and employment satisfaction. Taken together, these patterns suggest that education–employment mismatch is a broadly shared issue, but one that takes shape differently across national settings, depending on educational expansion, labour market structure, and institutional context.

### 5.4 Limitations and directions for future research

This study sheds light on the ways overeducation and vertical skill mismatch shape graduates’ employment outcomes in China, but several limitations should be noted. First, the cross-sectional design does not allow firm causal claims, and future research would benefit from longitudinal or panel data to capture how educational and vertical skill mismatches unfold over time. Second, the analysis is based on self-reported data. Although common method bias does not appear to pose a serious threat, respondents’ accounts may still be affected by recall error or social desirability. In addition, because unemployed and job-seeking respondents answered job-related items with reference to their most recent employment experience, their responses may involve retrospective reporting bias and may not fully capture their current employment situation. These respondents may also interpret vertical skill mismatch, skill utilization, and employment satisfaction differently from currently employed graduates. Future research should examine this issue more directly by conducting sensitivity analyses excluding unemployed respondents or by comparing employed and unemployed graduates in larger samples. Further studies could strengthen the evidence by incorporating additional sources, such as employer assessments or administrative records. Third, the use of convenience and snowball sampling may limit the representativeness of the sample and, therefore, the broader generalizability of the findings. Future research drawing on larger and more representative datasets would help test the robustness of the model across different regions and disciplines. Despite these limitations, the study provides a useful basis for further inquiry into graduate employability in changing labour markets.

## 6 Conclusion

This study examined the associations among overeducation, vertical skill mismatch, skill utilization, and employment outcomes among Chinese university graduates, with particular attention to whether skill utilization helps explain the relationship between mismatch and employment outcomes. The findings suggest that overeducation is associated with a higher likelihood of vertical skill mismatch, which is in turn related to fewer opportunities for skill utilization and lower levels of employment satisfaction and employment quality. Rather than proving a causal mechanism, these results provide associational evidence that is consistent with the proposed sequential mediation pathway.

By integrating human capital theory, job–skill matching theory, and social cognitive perspectives, the study contributes to a fuller understanding of graduate employability in mass higher education systems. The findings are consistent with the view that educational attainment is more likely to be associated with positive employment outcomes when graduates have access to jobs that allow them to use and further develop their skills. They also suggest that graduate employability should be understood not only in terms of educational credentials or employment status, but also in relation to the extent to which graduates’ skills are meaningfully utilized in the workplace.

From a policy perspective, the findings point to the importance of improving the alignment between higher education and labour market demand. Universities may benefit from strengthening curriculum relevance, experiential learning, and industry partnerships to reduce mismatch during the transition from education to work. Career services may also support students by helping them assess their skills, understand labour market expectations, and prepare for employment transitions. At the macro level, closer coordination among government agencies, higher education institutions, and employers may help create conditions in which graduates’ skills can be better recognized, developed, and used.

In conclusion, this study suggests that graduate employment challenges in China’s knowledge economy are associated not only with access to higher education, but also with the alignment between education, skills, and work. Because the study is based on cross-sectional survey data and non-probability sampling, the findings should be interpreted as evidence consistent with the proposed theoretical pathway rather than as causal proof. Future research should use longitudinal, panel, or nationally representative data to examine whether and how overeducation, vertical skill mismatch, and skill utilization shape graduates’ employment trajectories over time.

## Data Availability

The raw data supporting the conclusions of this article will be made available by the authors, without undue reservation.
