# Supplementary material for: Overeducation, vertical skill mismatch, and employment outcomes among Chinese university graduates: the mediating role of skill utilization
Source: Front Psychol. 2026 Jun 24;17:1859874. doi: 10.3389/fpsyg.2026.1859874 (PMC13341467; doi:10.3389/fpsyg.2026.1859874)
Supplement: Supplementary file 1 [file Supplementary_file_1.docx]

**Appendix A**

**Sampling Procedure and Institutional Distribution**

This appendix provides supplementary information on the sampling procedure and institutional distribution of respondents. Participants were recent university graduates recruited through a combination of convenience sampling and snowball sampling. Initial recruitment was conducted through university alumni networks, graduate employment-related online groups, and personal academic contacts. Eligible participants were invited to complete an anonymous online questionnaire and were encouraged to forward the survey link to other recent graduates who met the inclusion criteria. The final sample included 512 respondents from 15 universities across eastern, central, and western China.

**Table A1**

*Distribution of Respondents by University, Region, and Institutional Type*

| **University code** | **Region** | **Institutional type** | **Respondents** | **Percentage** |
| --- | --- | --- | --- | --- |
| U1 | Eastern China | Comprehensive university | 40 | 7.8% |
| U2 | Eastern China | Finance and economics university | 34 | 6.6% |
| U3 | Eastern China | Engineering / applied university | 31 | 6.1% |
| U4 | Eastern China | Normal university | 36 | 7.0% |
| U5 | Eastern China | Engineering-oriented university | 33 | 6.4% |
| U6 | Eastern China | Normal / comprehensive university | 31 | 6.1% |
| U7 | Central China | Comprehensive university | 38 | 7.4% |
| U8 | Central China | Engineering / applied university | 33 | 6.4% |
| U9 | Central China | Normal university | 29 | 5.7% |
| U10 | Central China | Finance and economics university | 30 | 5.9% |
| U11 | Central China | Engineering-oriented university | 31 | 6.1% |
| U12 | Western China | Comprehensive university | 42 | 8.2% |
| U13 | Western China | Engineering-oriented university | 36 | 7.0% |
| U14 | Western China | Normal university | 34 | 6.6% |
| U15 | Western China | Comprehensive / ethnic university | 34 | 6.6% |
| **Total** | **—** | **—** | **512** | **100.0%** |

Note. University names were anonymized as U1–U15.

**Appendix B**

Appendix B provides the additional statistical details requested during the review process. Tables B1–B4 report the full hierarchical regression results for both outcomes. Tables B5–B10 report the measurement and structural equation model in detail, including estimation equations, factor loadings, construct reliability and convergent validity, discriminant validity, structural path coefficients, indirect effects, and nested-model comparisons.

**Table B1. Hierarchical Regression Predicting Employment Satisfaction — Coefficients**

| **Predictor** | **B** | **SE** | **β** | **95% CI [LL, UL]** | **p** |
| --- | --- | --- | --- | --- | --- |
| **Step 1** | | | | | |
| const | 0.500 | 0.781 | — | [-1.033, 2.034] | 0.522 |
| gender_1 | 0.084 | 0.079 | 0.050 | [-0.070, 0.238] | 0.285 |
| major_2 | 0.183 | 0.102 | 0.093 | [-0.016, 0.383] | 0.071 |
| major_3 | 0.279** | 0.098 | 0.155 | [0.087, 0.472] | 0.004 |
| major_4 | 0.141 | 0.122 | 0.064 | [-0.099, 0.381] | 0.248 |
| univ_type_2 | -0.159 | 0.118 | -0.083 | [-0.390, 0.072] | 0.176 |
| univ_type_3 | -0.240* | 0.111 | -0.141 | [-0.458, -0.022] | 0.031 |
| age | 0.100** | 0.033 | 0.144 | [0.035, 0.165] | 0.003 |
| **Step 2** | | | | | |
| const | 1.177 | 0.779 | — | [-0.353, 2.707] | 0.131 |
| gender_1 | 0.085 | 0.077 | 0.051 | [-0.067, 0.236] | 0.272 |
| major_2 | 0.195 | 0.100 | 0.098 | [-0.001, 0.390] | 0.051 |
| major_3 | 0.278** | 0.096 | 0.155 | [0.090, 0.467] | 0.004 |
| major_4 | 0.157 | 0.120 | 0.071 | [-0.078, 0.392] | 0.190 |
| univ_type_2 | -0.113 | 0.116 | -0.059 | [-0.340, 0.115] | 0.331 |
| univ_type_3 | -0.194 | 0.109 | -0.114 | [-0.409, 0.020] | 0.076 |
| age | 0.095** | 0.032 | 0.136 | [0.031, 0.158] | 0.004 |
| OE | -0.187*** | 0.040 | -0.196 | [-0.266, -0.108] | <.001 |
| **Step 3** | | | | | |
| const | 2.322** | 0.735 | — | [0.877, 3.766] | 0.002 |
| gender_1 | 0.072 | 0.072 | 0.043 | [-0.069, 0.212] | 0.317 |
| major_2 | 0.189* | 0.093 | 0.096 | [0.007, 0.371] | 0.042 |
| major_3 | 0.244** | 0.089 | 0.136 | [0.069, 0.420] | 0.006 |
| major_4 | 0.143 | 0.111 | 0.065 | [-0.076, 0.362] | 0.200 |
| univ_type_2 | -0.126 | 0.108 | -0.065 | [-0.337, 0.085] | 0.242 |
| univ_type_3 | -0.186 | 0.102 | -0.109 | [-0.386, 0.014] | 0.068 |
| age | 0.081** | 0.030 | 0.117 | [0.022, 0.141] | 0.007 |
| OE | -0.082* | 0.039 | -0.086 | [-0.159, -0.006] | 0.036 |
| SM | -0.385*** | 0.043 | -0.367 | [-0.469, -0.300] | <.001 |
| **Step 4** | | | | | |
| const | 0.939 | 0.688 | — | [-0.414, 2.292] | 0.173 |
| gender_1 | 0.028 | 0.066 | 0.017 | [-0.102, 0.157] | 0.675 |
| major_2 | 0.175* | 0.085 | 0.088 | [0.008, 0.341] | 0.040 |
| major_3 | 0.208* | 0.082 | 0.116 | [0.047, 0.369] | 0.011 |
| major_4 | 0.159 | 0.102 | 0.072 | [-0.042, 0.359] | 0.121 |
| univ_type_2 | -0.113 | 0.099 | -0.059 | [-0.307, 0.080] | 0.251 |
| univ_type_3 | -0.165 | 0.093 | -0.097 | [-0.348, 0.018] | 0.077 |
| age | 0.069* | 0.028 | 0.099 | [0.014, 0.123] | 0.014 |
| OE | -0.013 | 0.037 | -0.014 | [-0.085, 0.058] | 0.712 |
| SM | -0.282*** | 0.041 | -0.268 | [-0.362, -0.201] | <.001 |
| SU | 0.377*** | 0.038 | 0.381 | [0.302, 0.452] | <.001 |

*Note.* N = 512. B = unstandardized coefficient; SE = robust standard error; β = standardized coefficient; CI = confidence interval; gender_1 = female (ref: male); major_2 = economics/management, major_3 = humanities/social sciences, major_4 = natural sciences (ref: engineering); univ_type_2 = mid-tier, univ_type_3 = lower-tier (ref: high-tier); OE = overeducation; SM = vertical skill mismatch; SU = skill utilization. * p < .05, ** p < .01, *** p < .001.

**Table B2. Hierarchical Regression Predicting Employment Satisfaction — Model Fit and Comparison**

| **Step** | **N** | **R²** | **Adj. R²** | **F** | **df1** | **df2** | **p(F)** | **ΔR²** | **F change** | **p(ΔF)** | **AIC** | **BIC** |
| --- | --- | --- | --- | --- | --- | --- | --- | --- | --- | --- | --- | --- |
| Step 1 | 512 | 0.082 | 0.070 | 6.46 | 7 | 504 | <.001 | — | — | — | 1237.3 | 1271.2 |
| Step 2 | 512 | 0.120 | 0.106 | 8.59 | 8 | 503 | <.001 | 0.038 | 21.64 | <.001 | 1217.7 | 1255.8 |
| Step 3 | 512 | 0.241 | 0.227 | 17.71 | 9 | 502 | <.001 | 0.121 | 79.87 | <.001 | 1144.1 | 1186.5 |
| Step 4 | 512 | 0.364 | 0.351 | 28.63 | 10 | 501 | <.001 | 0.123 | 96.57 | <.001 | 1055.9 | 1102.5 |

*Note.* N = 512. ΔR² and F change refer to the change from the immediately preceding step. AIC = Akaike information criterion; BIC = Bayesian information criterion. Smaller AIC/BIC values indicate better fit.

**Table B3. Hierarchical Regression Predicting Employment Quality — Coefficients**

| **Predictor** | **B** | **SE** | **β** | **95% CI [LL, UL]** | **p** |
| --- | --- | --- | --- | --- | --- |
| **Step 1** | | | | | |
| const | 1.030 | 0.858 | — | [-0.655, 2.715] | 0.230 |
| gender_1 | -0.022 | 0.086 | -0.012 | [-0.192, 0.147] | 0.798 |
| major_2 | 0.129 | 0.112 | 0.061 | [-0.090, 0.348] | 0.248 |
| major_3 | 0.170 | 0.108 | 0.088 | [-0.042, 0.381] | 0.115 |
| major_4 | 0.144 | 0.134 | 0.061 | [-0.120, 0.408] | 0.284 |
| univ_type_2 | 0.019 | 0.129 | 0.009 | [-0.235, 0.273] | 0.884 |
| univ_type_3 | -0.089 | 0.122 | -0.049 | [-0.329, 0.150] | 0.464 |
| age | 0.077* | 0.036 | 0.104 | [0.006, 0.148] | 0.035 |
| **Step 2** | | | | | |
| const | 1.699* | 0.859 | — | [0.011, 3.386] | 0.049 |
| gender_1 | -0.022 | 0.085 | -0.012 | [-0.188, 0.145] | 0.800 |
| major_2 | 0.140 | 0.110 | 0.066 | [-0.076, 0.356] | 0.203 |
| major_3 | 0.168 | 0.106 | 0.088 | [-0.040, 0.376] | 0.112 |
| major_4 | 0.160 | 0.132 | 0.067 | [-0.100, 0.419] | 0.227 |
| univ_type_2 | 0.065 | 0.128 | 0.032 | [-0.186, 0.316] | 0.611 |
| univ_type_3 | -0.044 | 0.121 | -0.024 | [-0.281, 0.192] | 0.713 |
| age | 0.072* | 0.036 | 0.097 | [0.002, 0.142] | 0.045 |
| OE | -0.185*** | 0.044 | -0.181 | [-0.272, -0.098] | <.001 |
| **Step 3** | | | | | |
| const | 2.662** | 0.838 | — | [1.017, 4.308] | 0.002 |
| gender_1 | -0.032 | 0.082 | -0.018 | [-0.193, 0.128] | 0.691 |
| major_2 | 0.136 | 0.105 | 0.064 | [-0.072, 0.343] | 0.199 |
| major_3 | 0.140 | 0.102 | 0.073 | [-0.060, 0.340] | 0.169 |
| major_4 | 0.148 | 0.127 | 0.062 | [-0.101, 0.397] | 0.244 |
| univ_type_2 | 0.054 | 0.123 | 0.026 | [-0.187, 0.294] | 0.662 |
| univ_type_3 | -0.037 | 0.116 | -0.021 | [-0.265, 0.190] | 0.748 |
| age | 0.061 | 0.034 | 0.082 | [-0.007, 0.128] | 0.078 |
| OE | -0.097* | 0.045 | -0.095 | [-0.184, -0.009] | 0.030 |
| SM | -0.324*** | 0.049 | -0.289 | [-0.420, -0.228] | <.001 |
| **Step 4** | | | | | |
| const | 1.038 | 0.779 | — | [-0.494, 2.569] | 0.184 |
| gender_1 | -0.084 | 0.074 | -0.047 | [-0.230, 0.062] | 0.259 |
| major_2 | 0.118 | 0.096 | 0.056 | [-0.070, 0.307] | 0.218 |
| major_3 | 0.098 | 0.093 | 0.051 | [-0.085, 0.280] | 0.293 |
| major_4 | 0.166 | 0.116 | 0.070 | [-0.061, 0.393] | 0.151 |
| univ_type_2 | 0.068 | 0.112 | 0.033 | [-0.151, 0.288] | 0.540 |
| univ_type_3 | -0.013 | 0.105 | -0.007 | [-0.220, 0.195] | 0.905 |
| age | 0.046 | 0.031 | 0.062 | [-0.016, 0.107] | 0.146 |
| OE | -0.016 | 0.041 | -0.015 | [-0.097, 0.066] | 0.704 |
| SM | -0.203*** | 0.046 | -0.181 | [-0.294, -0.112] | <.001 |
| SU | 0.443*** | 0.043 | 0.420 | [0.358, 0.528] | <.001 |

*Note.* N = 512. See the note to Table B1 for variable definitions. * p < .05, ** p < .01, *** p < .001.

**Table B4. Hierarchical Regression Predicting Employment Quality — Model Fit and Comparison**

| **Step** | **N** | **R²** | **Adj. R²** | **F** | **df1** | **df2** | **p(F)** | **ΔR²** | **F change** | **p(ΔF)** | **AIC** | **BIC** |
| --- | --- | --- | --- | --- | --- | --- | --- | --- | --- | --- | --- | --- |
| Step 1 | 512 | 0.029 | 0.016 | 2.17 | 7 | 504 | 0.036 | — | — | — | 1333.5 | 1367.4 |
| Step 2 | 512 | 0.062 | 0.047 | 4.13 | 8 | 503 | <.001 | 0.032 | 17.37 | <.001 | 1318.1 | 1356.3 |
| Step 3 | 512 | 0.137 | 0.121 | 8.83 | 9 | 502 | <.001 | 0.075 | 43.64 | <.001 | 1277.4 | 1319.8 |
| Step 4 | 512 | 0.285 | 0.271 | 19.99 | 10 | 501 | <.001 | 0.148 | 104.08 | <.001 | 1182.8 | 1229.4 |

*Note.* N = 512. ΔR² and F change refer to the change from the preceding step.

**Table B5. Measurement Model — Standardized Factor Loadings**

| **Construct** | **Indicator** | **B** | **SE** | **z** | **p** | **β (Std.)** |
| --- | --- | --- | --- | --- | --- | --- |
| **OE** | OE_1 | 1.000 | — | — | — | 0.828 |
|  | OE_2 | 0.935 | 0.054 | 17.23 | <.001 | 0.772*** |
|  | OE_3 | 0.970 | 0.055 | 17.59 | <.001 | 0.797*** |
| **SM** | SM_1 | 1.000 | — | — | — | 0.773 |
|  | SM_2 | 0.995 | 0.056 | 17.81 | <.001 | 0.780*** |
|  | SM_3 | 1.076 | 0.056 | 19.09 | <.001 | 0.834*** |
|  | SM_4 | 1.018 | 0.055 | 18.36 | <.001 | 0.803*** |
| **SU** | SU_1 | 1.000 | — | — | — | 0.773 |
|  | SU_2 | 1.006 | 0.056 | 17.81 | <.001 | 0.780*** |
|  | SU_3 | 0.947 | 0.055 | 17.17 | <.001 | 0.754*** |
|  | SU_4 | 1.009 | 0.056 | 17.89 | <.001 | 0.783*** |
|  | SU_5 | 0.933 | 0.057 | 16.29 | <.001 | 0.719*** |
| **ES** | ES_1 | 1.000 | — | — | — | 0.798 |
|  | ES_2 | 0.955 | 0.049 | 19.38 | <.001 | 0.791*** |
|  | ES_3 | 0.974 | 0.050 | 19.57 | <.001 | 0.797*** |
|  | ES_4 | 1.017 | 0.050 | 20.32 | <.001 | 0.821*** |
|  | ES_5 | 0.977 | 0.050 | 19.37 | <.001 | 0.790*** |

*Note.* N = 512. B = unstandardized loading; SE = standard error; β = standardized loading. The first indicator of each latent construct was fixed to 1 for identification, so its SE, z, and p are not estimated. Model fit (measurement model): χ²(113) = 130.23, p = 0.128, CFI = 0.996, TLI = 0.995, RMSEA = 0.017. *** p < .001.

**Table B6. Construct Reliability and Convergent Validity**

| **Construct** | **k** | **Cronbach's α** | **CR** | **AVE** | **√AVE** |
| --- | --- | --- | --- | --- | --- |
| **OE** | 3 | 0.841 | 0.841 | 0.639 | 0.799 |
| **SM** | 4 | 0.875 | 0.875 | 0.636 | 0.798 |
| **SU** | 5 | 0.873 | 0.874 | 0.581 | 0.762 |
| **ES** | 5 | 0.899 | 0.899 | 0.639 | 0.800 |

*Note.* N = 512. k = number of items; α = Cronbach's alpha; CR = composite reliability = (Σλ)² / [(Σλ)² + Σ(1 − λ²)]; AVE = average variance extracted = Σλ² / k. Recommended thresholds: α ≥ .70, CR ≥ .70, AVE ≥ .50. All four constructs meet these thresholds, indicating adequate internal consistency and convergent validity.

**Table B7. Discriminant Validity — Fornell-Larcker Criterion (Panel A) and Heterotrait-Monotrait Ratio (Panel B)**

**Panel A. Fornell-Larcker criterion (√AVE on diagonal, correlations off-diagonal)**

| **Construct** | **OE** | **SM** | **SU** | **ES** |
| --- | --- | --- | --- | --- |
| **OE** | **0.799** |  |  |  |
| **SM** | 0.303 | **0.798** |  |  |
| **SU** | -0.275 | -0.328 | **0.762** |  |
| **ES** | -0.213 | -0.419 | 0.501 | **0.800** |

**Table B8. Structural Equation Model — Path Coefficients with Bootstrap CIs**

| **Path** | **B** | **SE** | **β** | **95% CI [LL, UL]** | **p** |
| --- | --- | --- | --- | --- | --- |
| OE → SM | 0.317*** | 0.045 | 0.330 | [0.227, 0.410] | <.001 |
| OE → SU | -0.228*** | 0.051 | -0.235 | [-0.325, -0.127] | <.001 |
| SM → SU | -0.272*** | 0.048 | -0.269 | [-0.363, -0.178] | <.001 |
| SU → ES | 0.486*** | 0.051 | 0.486 | [0.390, 0.589] | <.001 |
| SU → EQ | 0.534*** | 0.053 | 0.481 | [0.429, 0.645] | <.001 |
| SM → ES | -0.277*** | 0.044 | -0.274 | [-0.360, -0.198] | <.001 |
| SM → EQ | -0.186*** | 0.047 | -0.166 | [-0.285, -0.083] | <.001 |
| OE → ES | 0.002 | 0.045 | 0.002 | [-0.091, 0.090] | 0.968 |
| OE → EQ | 0.006 | 0.049 | 0.006 | [-0.091, 0.108] | 0.902 |

*Note.* N = 512. B = unstandardized path coefficient; SE = standard error; β = standardized path coefficient; CI = bias-corrected percentile bootstrap confidence interval (5,000 resamples). Overall model fit: χ²(83) = 107.96, p = 0.034, CFI = 0.993, TLI = 0.992, RMSEA = 0.024. * p < .05, ** p < .01, *** p < .001.

**Table B9. Decomposition of Direct, Indirect, and Total Effects (Bootstrap, 5,000 Resamples)**

| **Effect** | **B** | **Boot SE** | **95% CI [LL, UL]** | **p** |
| --- | --- | --- | --- | --- |
| **Outcome: Employment Satisfaction (ES)** | | | | |
| **Specific indirect effects** | | | | |
| OE → SM → SU → ES (serial) | -0.042 | 0.010 | [-0.064, -0.025] | <.001 |
| OE → SM → ES | -0.088 | 0.019 | [-0.130, -0.054] | <.001 |
| OE → SU → ES | -0.111 | 0.028 | [-0.168, -0.060] | <.001 |
| SM → SU → ES | -0.132 | 0.026 | [-0.186, -0.084] | <.001 |
| **Total indirect** | | | | |
| Total indirect OE → ES | -0.240 | 0.035 | [-0.312, -0.177] | <.001 |
| **Direct and total effects** | | | | |
| Direct OE → ES | 0.002 | 0.046 | [-0.091, 0.090] | 0.963 |
| Total OE → ES | -0.239 | 0.050 | [-0.342, -0.144] | <.001 |
| Direct SM → ES | -0.277 | 0.041 | [-0.360, -0.198] | <.001 |
| Total SM → ES | -0.409 | 0.043 | [-0.493, -0.324] | <.001 |
| **Outcome: Employment Quality (EQ)** | | | | |
| **Specific indirect effects** | | | | |
| OE → SM → SU → EQ (serial) | -0.046 | 0.011 | [-0.069, -0.027] | <.001 |
| OE → SM → EQ | -0.059 | 0.020 | [-0.101, -0.024] | <.001 |
| OE → SU → EQ | -0.122 | 0.030 | [-0.183, -0.066] | <.001 |
| SM → SU → EQ | -0.145 | 0.029 | [-0.204, -0.091] | <.001 |
| **Total indirect** | | | | |
| Total indirect OE → EQ | -0.227 | 0.034 | [-0.298, -0.162] | <.001 |
| **Direct and total effects** | | | | |
| Direct OE → EQ | 0.006 | 0.050 | [-0.091, 0.108] | 0.887 |
| Total OE → EQ | -0.221 | 0.051 | [-0.320, -0.123] | <.001 |
| Direct SM → EQ | -0.186 | 0.051 | [-0.285, -0.083] | <.001 |
| Total SM → EQ | -0.331 | 0.054 | [-0.434, -0.223] | <.001 |

*Note.* N = 512. B = unstandardized effect; Boot SE = bootstrap standard error; CI = bias-corrected percentile bootstrap 95% confidence interval based on 5,000 resamples. An effect is considered statistically significant when the 95% CI does not contain zero. The serial indirect effect OE → SM → SU → outcome is the main mediation pathway hypothesized in H4.

**Table B10. Model Comparison — Proposed Model vs. Alternative Specifications**

| **Model** | **χ²** | **df** | **Δχ²** | **Δdf** | **p(Δχ²)** | **CFI** | **TLI** | **RMSEA** | **AIC** | **BIC** |
| --- | --- | --- | --- | --- | --- | --- | --- | --- | --- | --- |
| M1. Proposed serial mediation (with direct paths) | 107.96 | 83 | — | — | — | 0.993 | 0.992 | 0.024 | 73.58 | 230.40 |
| M2. Parallel mediation (SM → SU path dropped) | 139.66 | 84 | 31.70 | 1 | <.001 | 0.985 | 0.981 | 0.036 | 71.45 | 224.03 |
| M3. Full mediation (OE → outcomes direct paths dropped) | 107.97 | 85 | 0.02 | 2 | 0.992 | 0.994 | 0.992 | 0.023 | 69.58 | 217.92 |
| M4. Reversed mediator order | 107.96 | 83 | — | — | — | 0.993 | 0.992 | 0.024 | 73.58 | 230.40 |

*Note. N = 512.* Model specifications: M1 — OE → SM, SU ~ SM + OE, outcomes ~ SU + SM + OE (proposed serial mediation with residual direct paths). M2 — same as M1 but with the SM → SU path fixed to zero (parallel mediation). M3 — same as M1 but with the OE → ES and OE → EQ direct paths fixed to zero (full mediation). M4 — OE → SU, SM ~ SU + OE, outcomes ~ SM + SU + OE (reversed mediator order). Δχ² and Δdf compare each nested model with M1; a significant Δχ² indicates the simpler model fits significantly worse. M2 fits significantly worse than M1 (Δχ² = 31.70, Δdf = 1, p < .001), supporting the serial pathway. M3 does not fit significantly worse than M1 (Δχ² = 0.02, Δdf = 2, p > .99), indicating that the direct OE → outcome paths are statistically redundant given the mediating chain (consistent with full serial mediation). M4 swaps SM and SU without changing the number of estimated parameters and is therefore statistically equivalent to M1 (identical χ², df, and global fit indices); the theoretical ordering must be defended on conceptual grounds (see Section 3.5 of the manuscript).

**Table B11**

*Comparison of Key Study Variables Between Horizontally Matched and Horizontally Mismatched Graduates*

| **Variable** | **Horizontally matched**  **(*n* = 283)** | **Horizontally mismatched**  **(*n* = 229)** | **Mean difference**  **(mismatched − matched)** | **Welch *t*** | ***p*** | **Cohen's *d*** |
| --- | --- | --- | --- | --- | --- | --- |
| Overeducation | 3.17 (0.86) | 3.24 (0.88) | 0.07 | 0.90 | .369 | 0.08 |
| Vertical Skill Mismatch | 2.87 (0.72) | 3.08 (0.86) | 0.21 | 3.01 | .003 | 0.27 |
| Skill Utilization | 3.26 (0.82) | 2.96 (0.84) | −0.29 | −3.96 | < .001 | −0.35 |
| Employment Satisfaction | 3.01 (0.83) | 2.75 (0.81) | −0.26 | −3.60 | < .001 | −0.32 |
| Employment Quality | 3.19 (0.84) | 2.56 (0.83) | −0.63 | −8.48 | < .001 | −0.75 |
| Employment Quality excl. match | 3.02 (1.06) | 2.66 (1.06) | −0.36 | −3.86 | < .001 | −0.34 |

*Note.* Values for the first five rows are presented as M (SD) on a 1–5 metric. The composite employment quality (EQ) score was computed as the average of four indicators (monthly income, job stability, employment status, major–job match) following the aggregation rule reported in Section 3.3.4. *Employment Quality excl. match* was computed as (4 × EQ − EQ_match_) ⁄ 3, removing the contribution of the major–job match indicator from the composite while preserving the original aggregation procedure. Group sizes: *n* = 283 matched, *n* = 229 mismatched. Welch's two-sample *t* tests were used because group variances were not assumed equal. *p* values are two-tailed. Cohen's *d* was computed as (M_mismatched_ − M_matched_) / SD_pooled_; conventional benchmarks (Cohen, 1988): |*d*| ≈ 0.20 (small), 0.50 (medium), 0.80 (large).

**Supplementary Appendix C. Adapted Questionnaire Items and Coding Procedures**

**Note.** The questionnaire was administered in Chinese. The English version presented in this appendix is provided for reporting purposes. Some items were adapted from previously published scales or measurement frameworks and modified for the Chinese graduate employment context. To avoid reproducing previously published instruments verbatim without explicit permission, this appendix reports the adapted questionnaire information and coding procedures used in the present study rather than the original source scale items in full.

**Table C1**

*Measurement Information and Coding Procedures*

| **Construct** | **Item codes** | **Measurement information** | **Response scale / coding** | **Measurement source** |
| --- | --- | --- | --- | --- |
| Perceived overeducation | OE1-OE3 | Adapted items assessing the extent to which respondents perceived their current job as requiring a lower level of education than they had attained. Higher scores indicate stronger perceived overeducation. | 1 = strongly disagree to 5 = strongly agree | Adapted from Verhaest and Omey (2006) |
| Vertical Skill Mismatch | SM1-SM4 | Respondents rated both job-required skill levels and self-assessed skill levels across analytical ability, communication, teamwork, and problem solving. Vertical skill mismatch was calculated based on the discrepancy between self-assessed skill level and job-required skill level. Higher scores indicate greater surplus skills. | 1-5 scale; discrepancy-based coding | Based on Allen and Van der Velden (2001) |
| Skill utilization | SU1-SU5 | Adapted items assessing the extent to which graduates were able to use and further develop their acquired skills in their current jobs. Higher scores indicate higher skill utilization. | 1 = strongly disagree to 5 = strongly agree | Adapted from García-Aracil and Van der Velden (2008) |
| Employment satisfaction | ES1-ES5 | Adapted items assessing graduates' overall affective and evaluative satisfaction with their current job. Higher scores indicate higher employment satisfaction. | 1 = strongly disagree to 5 = strongly agree | Adapted from Brayfield and Rothe (1951) |
| Employment quality | EQ1-EQ4 | A composite observed outcome constructed from monthly income, job stability, employment status, and major-job match. Higher scores indicate better employment quality. | Indicators were coded or rescaled so that higher values consistently indicated better employment quality; the composite was calculated by averaging the four indicators. | Constructed in the present study based on graduate employment indicators |

**Note.** OE = perceived overeducation; SM = vertical skill mismatch; SU = skill utilization; ES = employment satisfaction; EQ = employment quality.
